# Physiotherapy Rehabilitation for Osteoporotic Vertebral Fracture (PROVE): study protocol for a randomised controlled trial

**DOI:** 10.1186/1745-6215-15-22

**Published:** 2014-01-14

**Authors:** Karen L Barker, Muhammad K Javaid, Meredith Newman, Catherine Minns Lowe, Nigel Stallard, Helen Campbell, Varsha Gandhi, Sallie Lamb

**Affiliations:** 1 NIHR – BRU, Nuffield Department of Orthopaedics, Rheumatology and Musculoskeletal Sciences, University of Oxford, Oxford OX3 7LD, UK; 2 Physiotherapy Research Unit, Nuffield Orthopaedic Centre, Oxford University Hospitals NHS Trust, Windmill Road, Oxford OX3 7HE, UK; 3 Division of Health Sciences, Warwick Medical School, University of Warwick, Coventry CV4 7AL, UK; 4 Health Economics Research Centre, Department of Public Health, University of Oxford, Oxford OX3 7LF, UK

**Keywords:** Randomised controlled trial, Osteoporosis, Rehabilitation, Vertebral fracture

## Abstract

**Background:**

Osteoporosis and vertebral fracture can have a considerable impact on an individual’s quality of life. There is increasing evidence that physiotherapy including manual techniques and exercise interventions may have an important treatment role. This pragmatic randomised controlled trial will investigate the clinical and cost-effectiveness of two different physiotherapy approaches for people with osteoporosis and vertebral fracture, in comparison to usual care.

**Methods/Design:**

Six hundred people with osteoporosis and a clinically diagnosed vertebral fracture will be recruited and randomly allocated to one of three management strategies, usual care (control - A), an exercise-based physiotherapy intervention (B) or a manual therapy-based physiotherapy intervention (C). Those in the usual care arm will receive a single session of education and advice, those in the active treatment arms (B + C) will be offered seven individual physiotherapy sessions over 12 weeks. The trial is designed as a prospective, adaptive single-blinded randomised controlled trial. An interim analysis will be completed and if one intervention is clearly superior the trial will be adapted at this point to continue with just one intervention and the control. The primary outcomes are quality of life measured by the disease specific QUALLEFO 41 and the Timed Loaded Standing test measured at 1 year.

**Discussion:**

There are a variety of different physiotherapy packages used to treat patients with osteoporotic vertebral fracture. At present, the indication for each different therapy is not well defined, and the effectiveness of different modalities is unknown.

**Trial registration:**

Reference number ISRCTN49117867.

## Background

Each year 25,000 people in the UK have vertebral fractures related to their osteoporosis and many are referred for physiotherapy to help them recover after their fracture. Osteoporosis and vertebral fracture can have a considerable impact on an individual’s health-related quality of life (QoL) due to pain, limitations in activity, social participation and altered mood [1,2]. Vertebral fractures are closely related to increased thoracic kyphosis, which, along with a loss of lumbar lordosis, is linked to increased spinal loading and back extensor muscle weakness. This can lead to an increased risk of further fracture [3,4]. Hyperkyphotic posture is also associated with increased back pain and balance disturbance with a subsequent increased risk of falls and fractures as a result of falling [5,6].

Physiotherapy includes a variety of treatment options, such as exercise programmes or ‘hands on’ treatments such as massage and mobilisations. There is increasing evidence that physiotherapy interventions that address pain and physical impairments may have an important role in improving QoL and reducing the fracture risk in people with osteoporotic vertebral fractures. However, we do not know which type of physiotherapy is most helpful, the cost of treatment to the National Health Service (NHS), or what patients think of their treatment.

### Evidence for manual therapies

#### Manual mobilisation

Traditionally, physiotherapists use manual mobilisation in the management of back pain. However, evidence for the effectiveness and safety of manual mobilisations in the management of thoracic hyperkyphosis in elderly people is limited. Some physiotherapy guidelines caution against using spinal mobilisation in individuals with osteoporosis. High velocity spinal manipulation techniques are contraindicated [7] and concerns about the use of low velocity spinal mobilisation techniques have been expressed. However, recent practice surveys, case reports and two randomised controlled trials (RCTs) suggest these techniques can be used safely [8-11]. No serious adverse events were reported in either RCT of low velocity spinal mobilisation. Both RCTs have small sample sizes, short-term follow-up and have tested a combined treatment protocol of exercise and manual therapy including postural taping. These factors make it difficult to draw confident inference about the safety and effectiveness of the various treatment methods [8,9].

#### Postural taping

Postural taping uses tape applied to the skin to provide increased proprioceptive feedback about postural alignment, improve thoracic extension, reduce pain and facilitate postural muscle activity and balance [6,8,9,12].

### Evidence for exercise interventions

A number of systematic reviews and meta-analyses report the positive effects of exercise on bone mineral density (BMD), muscle strength, QoL and falls and fractures in men and women with osteoporosis or low BMD [13-16]. It is known that an osteoporotic vertebral fracture leads to axial posture deformity which can increase both the fear and the actual risk of falling. Strategies to address falling are an important component of any treatment programme for this patient group [5,6,17,18]. A number of RCTs have found that a combined balance and progressive strength training programme produced the best results in terms of maintaining leg strength, balance, BMD and physical function compared to balance or strength training alone [14,19-21].

RCTs of exercise interventions in people with vertebral osteoporosis also report benefits of reduced pain and improved QoL, strength and balance [22-28]. Interventions range from simple back extension exercises to a variety of general weight-bearing exercise, balance activities, stretches and combined upper limb, trunk and lower limb strengthening. The interventions were delivered in a class format [26-28], as a home programme [23,25] or as a combination of physiotherapist-led along with home exercise programmes [28]. There was no evidence that any of these delivery options was superior to another.

Overall, there is some evidence that both manual therapy and exercise interventions can be beneficial for this patient group. A limited number of studies [8-12] provide strong support for the use of manual therapies but these are inadequately powered. There is some higher quality evidence available to support exercise prescription for individuals with osteoporosis but only a few studies examine exercise in osteoporotic populations with vertebral fracture [22,24,28]. Of these, only a small number combine weight-bearing, strength and balance activities and, to date, none have included men. Whether exercise therapy is more effective than manual therapy is not known and further information is needed about the longer term outcomes of either intervention.

### Objectives

This paper describes the trial protocol for a large, pragmatic RCT to assess the effects of a physiotherapy intervention based on exercise or manual therapy compared to usual care for people with osteoporosis and a clinically diagnosed vertebral fracture.

The secondary objectives are: 1) to compare the effects of manual therapy with exercise therapy; 2) to investigate the acceptability and adherence to the physiotherapy programmes for both patients and therapists; 3) to conduct a parallel health economic analysis to assess the cost effectiveness of the different treatment strategies from an NHS, Social Services and patients’ perspective; and 4) to conduct a nested qualitative study to explore the experiences and views of people with osteoporosis and vertebral fracture regarding their treatment, their perceptions regarding the appropriateness and acceptability of the interventions and to explore the factors influencing their adherence to the intervention programmes.

## Methods/Design

### Trial design

The trial will be a prospective, multi-centre assessor-blinded, three-arm RCT with a nested qualitative study and an adaptive design. Patients will be randomised between three arms: usual care (control) group (A), manual therapy (B) and exercise therapy (C). Those in the usual care arm will receive a single session of education and advice, and those in the active treatment arms (B + C) will be offered up to seven individual physiotherapy sessions over 12 weeks. Clinical assessment will be performed at baseline (week 0), 4 months and 12 months, with postal questionnaires about QoL administered at 6 and 9 months. An interim analysis will be conducted once 75 participants are recruited to each arm and have completed their 4 month follow-up. Following this interim analysis the study may be adapted. If both intervention arms are sufficiently promising and sufficiently similar the study will not be adapted and recruitment will continue into both intervention arms. If one arm, manual therapy or exercise therapy, is not sufficiently promising relative to control or sufficiently similar to the other intervention arm, this arm will be dropped from the study and the study will be adapted to continue as a two arm RCT with participants randomised between the control and remaining intervention arm. If neither arm is sufficiently superior to the usual care arm the trial will be stopped.

**Figure 1:**
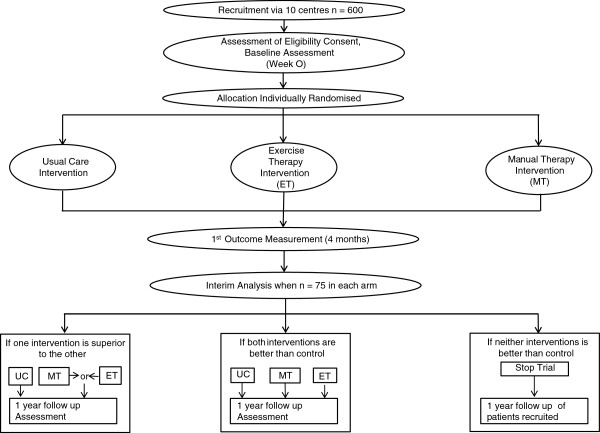
Study flow chart.

The protocol conforms to CONSORT guidelines for non-pharmacological studies [29] (Figure 1).

### Ethics approval

The study protocol was approved by South Central Research Ethics Committee [Reference 12/SC/0411]. Oxford University is the sponsor. The trial is registered with the International Standard Randomised Controlled Trials database ISRCTN reference number 49117867.

### Trial status

Recruitment started in **November 2013**.

### Participants

Six hundred patients will be recruited from 10 or more centres across the UK. Men and women with osteoporosis who have had a least one symptomatic vertebral fracture will be eligible for inclusion if they meet the following criteria.

### Inclusion criteria

1. Willing and able to give informed consent for participation in the study.

2. Diagnosis of primary osteoporosis confirmed by radiograph or DEXA scan (−2.5 SD below young adult mean) at lowest lumbar level.

3. At least one previous vertebral fracture.

4. Men and women aged 18 years or older.

5. Female participants to be postmenopausal; that is, no period in the last 2 years.

6. All should be able to walk at least 10 metres independently with or without an aid and be able to understand and participate in a physiotherapy programme.

### Exclusion criteria

Individuals may not enter the study if they have any condition which might make participating in the physiotherapy or exercise regimes unsafe or confound results. People with the following will therefore be excluded:

1. Severe unstable cardiovascular or pulmonary disease.

2. Significant psychiatric or neurological conditions.

3. Bone loss secondary to other metabolic bone disorders or disease will be excluded (for example, rheumatoid arthritis, cancer, osteomalacia).

4. Individuals whose primary problem is back pain with pain radiating into the lower limb.

5. Individuals who have had a vertebroplasty, facet joint injection or any physical therapy (for example, chiropractic, osteopathy or physiotherapy treatment) for back pain in the previous 12 weeks. However, individuals who have had back pain and any of these treatments prior to this period will be eligible.

### Procedures

#### Recruitment

Potential participants will be approached by clinicians during their routine clinic attendance. Clinicians treating potentially eligible participants for the trial will introduce the study, hand out an invitation pack and obtain consent for a Physiotherapy Rehabilitation for Osteoporotic Vertebral Fracture (PROVE) trained researcher to make contact 3 to 7 days later. Participants consenting to enrol in the study will undergo a baseline assessment; following this the assessor will telephone the independent randomisation centre.

#### Randomisation, blinding and allocation concealment

Randomisation will be performed by an independent statistician and implemented by the central telephone registration and randomisation service at the Warwick Clinical Trials Unit. Staff will register patients after confirming eligibility, consent and baseline assessment, thus ensuring allocation concealment.

Baseline and follow-up assessments will be performed by a blinded research physiotherapist. These staff will not be involved in delivering the treatment interventions. All data will be entered by a data entry assistant to ensure the research physiotherapists remain blind to treatment assignment. All study personnel involved in data entry, management and analysis will be blinded until the final analysis is complete. By virtue of the design it is not possible to blind the participants or the physiotherapists providing the treatment interventions [30].

After randomisation the Clinical Trials Unit will inform the research therapist at each site of a participant’s treatment allocation, and they will liaise with appropriate clinical staff to provide the correct intervention.

### Baseline assessment

After participants have been assessed for eligibility and consent has been gained, baseline assessment will be carried out. Questionnaires and physical assessment will be completed (both participant and assessor will be unaware of allocation at this appointment). The baseline measures are summarised in Table 1.

**Table 1:** Summary of outcomes and assessment schedule

**Tests**	**Administered by**	**Week 0**	**Month 4**	**Month 6**	**Month 9**	**Month 12**
Functional Co-morbidities Index	Self	*√*				
QUALEFFO 41	Self	*√*	*√*	By post	By post	*√*
EQ-5D-5 L	Self	*√*	*√*	By post	By post	*√*
Physical Activity Scale for the Elderly	Self	*√*	*√*	By post	By post	*√*
Timed Load Standing	Assessing therapist	*√*	*√*			*√*
Flexicurve ruler	Assessing therapist	*√*	*√*			*√*
The Short Performance Physical Battery	Assessing therapist	*√*	*√*			*√*
The Functional Reach test	Assessing therapist	*√*	*√*			*√*
6 minute walk test	Assessing therapist	*√*	*√*			*√*
10 point visual analogue scale	Self	*√*	*√*			*√*

### Outcome measures

There are two primary outcomes for this study: one measure of QoL and one measure of physical function.

1. The QUALEFFO 41 is a disease-specific measure of health related QoL applicable to patients with established vertebral osteoporosis. It is a self-administered questionnaire that provides scores on five domains; pain, physical function, social function, general health perception, mental performance, and a total score. It is validated and reliable and has been shown to be responsive in clinical trials of physiotherapy treatment [31]

2. The Timed Loaded Standing (TLS) test assesses back extensor muscle endurance [32]. Based upon previous literature [9], a 2.6 second change in the TLS test would be clinically significant for a change between the groups.

The study sample size gives adequate power to detect a treatment effect in both the QUALEFFO 41 and TLS as described more fully below.

### Secondary outcomes

There are a number of outcomes included seeking to assess a number of different aspects of symptoms, function and activity. We will be piloting the package, and if the burden of measurement is too great, some measures will be dropped and the protocol amended.

The Functional Co-morbidities Index will be completed as other diseases are likely to be present in this older population which might affect physical outcomes [33].

Spinal curvature and flexed posture will be described by recording height (cm) and thoracic kyphosis. Thoracic kyphosis will be measured using a flexicurve ruler with the patient in a standing position to calculate an index and angle of kyphosis [34].

Other outcomes include measures of balance, mobility and physical activity. Each test is reliable and valid, has been used with older, community dwelling adults and shown to be responsive in previous rehabilitation studies.

•The Short Performance Physical Battery will be used to assess lower extremity physical function [35]. Poor performance is predictive of future disability, hospitalisation and care needs.

•The Functional Reach Test will be used to specifically evaluate standing balance and to act as a predictor of falls risk [36].

•A 6 minute walk at self-selected speed over a 30 metre course will be used to measure exercise endurance; an important parameter of functional, community mobility [37].

•The Physical Activity Scale for the Elderly is a short, self-administered questionnaire to assess activity in the past week [38].

•The EuroQol EQ-5D-5 L is a short, generic measure of QoL and will be completed to facilitate the assessment of cost-effectiveness and also to give a comparison with other clinical conditions [39].

•Participants will be asked to specifically rate back pain on activity and at rest using a 10 point visual analogue scale.

•Information about fractures and falls will be collected using a standardised definition and a prospective participant completed event calendar. These will be mailed to participants monthly during the year of the study and participants will be telephoned by the trial co-ordinator to promote adherence and to check this information is captured precisely [40].

### Follow-up data collection

Follow-up data collection will be by face-to-face clinical assessment at 4 and 12 months.

### Interventions

Treatments are standardised, but it is considered important to allow therapists to personalise treatments as appropriate. For example, therapists will be able to omit or adjust the intensity of any technique or exercise to reflect an individual participant’s capabilities and their progress.

#### Best practice usual care

Currently, relatively few patients are referred for formal physiotherapy for an osteoporotic vertebral fracture. Therefore, a single education session will form the usual care arm. The education will be general advice about osteoporosis, and lifestyle choices to promote bone health in line with the information available from the National Osteoporosis Society.

#### Active interventions

Participants in each active intervention group will be offered up to seven individual physiotherapy sessions over 12 weeks. A 12-week programme was chosen to allow time to progress the treatment intensity and achieve gains in strength and mobility [9,25]. This pragmatic regime allocates equivalent physiotherapist contact time to participants in each intervention group and broadly reflects current outpatient physiotherapy resources within the NHS. Alongside individual sessions, participants in each intervention group will receive education about osteoporosis and general advice about exercise.

##### Manual therapy intervention

Manual therapies will include low velocity spinal mobilisation performed without discomfort [8-10] and soft tissue mobilisation to erector spinae, rhomboids and upper trapezius muscles [9]. Postural taping will be used once weekly and, if tolerated, it will be worn continuously for 3 days for the first 4 weeks. It will be applied to create gentle skin traction and sensory feedback about posture when the individual moves into flexion [8,9,12]. The home programme will be a passive stretch that promotes thoracic extension for 15 minutes per day [4].

##### Exercise intervention

**Figure 2:**
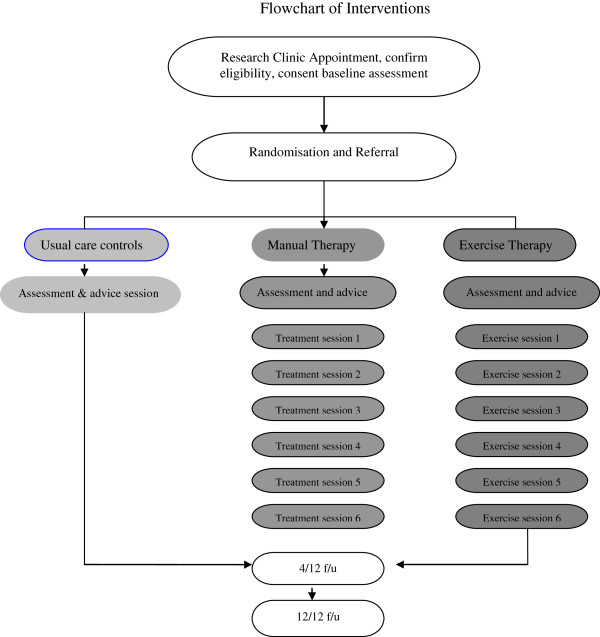
**Flowchart of interventions.** 4/12 f/u, 4- and 12-month follow-up.

The exercise programme will include active stretches, progressive balance and strength training and low to moderate intensity weight-bearing aerobic activity (for example, walking). Exercises will be practised in the treatment sessions and continued in the home programme. The Rating of Perceived Exertion scale will be used to set the initial load of strength exercises and walking duration at a self-perceived moderate level of effort, to facilitate monitoring and allow structured progression. Participants will be asked to include short sessions of exercise within daily life, aiming to achieve a total of 60 minutes of exercise per day, three to five times a week depending on ability (for example, a 30 minute walk along with 30 minutes of stretches, balance and strengthening exercises [28]). Stretches will promote spinal extension, shoulder flexion, hip extension and ankle dorsiflexion [28]. Specific trunk extension and lumbar stabilisation exercises will be included [4,22,23]. There will also be upper body and lower body strengthening exercises [22,28] and specific balance exercises such as single leg standing [21,26]. Wherever possible exercises will use body weight and gravity to provide resistance and balance related activities to the programme (for example, sit to stand will be encouraged to increase proprioceptive input [20]). We will use a series of educational and motivational strategies to foster compliance: clinician-patient goal setting, the provision of an exercise diary with a log sheet to record sessions, along with a scheduled telephone call to support practice [41]. Completion of home practice will be monitored through exercise diaries and compliance will be defined as 60% completion. Participants undergoing the interventions will be encouraged to continue their home exercises at the end of the active phase of the trial. Figure 2 provides an overview of the interventions in each trial arm.

### Adherence

Compliance with treatment will be defined as attending at least four treatment sessions. The number of physiotherapy visits and content of treatment sessions will be recorded using both therapist-completed treatment logs and patient exercise and participation diaries. Compliance of the physiotherapists to the treatment protocol will be assessed and monitored by analysis of the treatment logs and by site visits.

### Quality monitoring

There will be initial training provided to all PROVE trial assessors and clinicians involved in delivering the intervention at all sites. Once the training has been completed the following procedures are in place to promote consistency and high quality trial procedures across all sites:

•A member of the PROVE team will observe each assessor perform one of their first assessments to ensure they take place as per protocol. Repeat visits will be undertaken should any concerns arise until reliable and valid assessments occur.

•Within 2 weeks copies of all assessment forms will be sent to the trial co-ordinator for checking to identify any issues concerning missing data or poorly completed forms. Any issues or concerns will be discussed with individual assessors.

•A member of the PROVE team will observe each clinician perform one of their first treatments to ensure all treatments adhere to the protocol.

•Clinicians will be asked to complete a treatment log for each attendance, providing an approximate estimation of the time spent on key intervention components and detailing and explaining any deviations from the protocol.

•A member of the PROVE team will check each site’s trial master file and meet with researchers and clinicians from each site on an annual basis (or more frequently should this be necessary). During these meetings, feedback will be sought regarding trial procedures and the perceived acceptability of the intervention. Feedback will be documented and a copy of the minutes of the meeting will be returned to each site.

### Health economics

An economic evaluation is designed as an integral part of the trial and will take the form of a cost-utility analysis conducted from an NHS and Social Services perspective. Health outcomes will be expressed as quality-adjusted life years (QALYs) by combining survival and EuroQol EQ-5D data collected from each participant to 12 months. The cost to the health service of providing current practice and each type of physiotherapy intervention will be estimated during the trial, and participants will provide information to 12 months on healthcare contacts (for example, visits to the General Practitioner, outpatient clinics, and inpatient admissions). Care provided by Social Services will be documented as well as any out of pocket costs incurred by participants.

### Qualitative study

The qualitative study will help to provide a picture of the issues facing participants with osteoporosis and vertebral fracture who participate in the treatment interventions arms of the study. Semi-structured interviews will be conducted by a researcher experienced in the design, collection and analysis of qualitative data. Specific topics covered will be exercising with osteoporosis (including adherence issues), and participant experience of the PROVE trial. We will interview enough participants to ensure we are confident that theoretical sufficiency will be achieved [42]. Purposive sampling will be used to achieve a sample which includes female and male patients, thoracic and lumbar single/multiple vertebral fracture patients and patients of varying activity levels. Fracture site, number of fractures and activity levels are known to influence outcome and, since the majority of research regarding osteoporotic patients’ QoL has previously been undertaken with women, it is considered important to capture the views of male participants within the current study.

Interviews will be conducted either in the hospital or participants homes following the completion of the intervention and the 4-month follow-up appointment. The development of the interview schedule will be iterative and the questions asked may develop and change as the interviews are conducted and analysed [43]. All interviews will be digitally recorded and fully transcribed for analysis. Analysis will utilise Smith’s experiential approach of Interpretative Phenomenological Analysis. A feature of Interpretative Phenomenological Analysis is that the first steps of analysis begin early in the research process with initial data coding and is simultaneous to data collection. Initial analysis of each interview will be carried out as soon after its completion as possible following the guidelines set out by Smith [43].

One in five of the interviews will be coded independently by a second member of the research team with experience of qualitative research to provide a different perspective on the coding. The research team will discuss the development of themes as the research progresses, once again with the aim of providing a different perspective and enhancing the development of themes.

### Data and statistical analysis

#### Interim analysis decision rules

The decision to adapt the trial following the interim analysis and whether to drop one of the treatment arms, or to continue, will be made by the Trial Steering Committee based upon the recommendations of the independent data monitoring committee (IDMC). The IDMC will base their decision on the data from the interim analysis, using the rules below, together with data on adverse events, falls and further fracture history. An interim analysis will be conducted when the 16-week follow-up data are available for 75 patients per treatment arm. The aim of this interim analysis is to terminate either the manual therapy or exercise therapy arm if it appears to be poorly performing relative to the other intervention or to the control, or to terminate the trial completely if both intervention arms appear to be performing poorly relative to the control.

Although the integrity of the trial in terms of type I error control does not require pre-specification of the decision rule to be used at the interim analysis, as a guideline for the IDMC the interim analysis decision rule will be based on comparison of the estimated mean change from baseline in the QUALEFFO score for each of the three study arms as follows:

a) If the mean change from baseline of the QUALEFFO score for an intervention arm is not more than 0.5 points greater than that for the control arm, that intervention arm will be dropped from the study. Note that under this rule both intervention arms might be dropped, in which case the study would stop due to futility.

b) If the mean change from baseline of the QUALEFFO score for one intervention group is more than 2 points higher than for the other group, the intervention with the lower mean change from baseline will be dropped from the study.

#### Sample size

The initial sample size calculation is based on a traditional approach to a three-arm trial. We wish to detect a standardised effect of 0.4 in the QUALEFFO; at 80% power and an alpha of 0.05 we would require 180 to 200 participants in each arm or 540 to 600 people (we will use 600 people as the upper limit).

A simulation study was conducted to estimate the power of the study assuming the interim decision rules above were used. The sample size for the interim analysis was chosen to be 75 per arm. This ensures that the power is high while the probability of continuing with an ineffective treatment is sufficiently low. If the true (unknown) treatment effect for an intervention is equal to the control, the probability of dropping that intervention at the interim analysis is approximately 73%. If neither intervention is truly superior to the control, the probability of stopping the entire study at the interim analysis is approximately 60%. Based on this interim analysis sample size the standard error of the estimated difference between the intervention arms and the control arm at the interim analysis will be 0.82.

In the adaptive design of the type proposed, the power of the study may be defined as the probability, given a truly effective intervention, that the intervention remains in the trial at the interim analysis and leads to a significant result in comparison to the control in the final analysis. The specified sample size gives 94% power if the better of the two intervention arms has a true standardised treatment effect of 0.4.

### Statistical analysis

We will report according to CONSORT standards.

Regression models will be used to estimate the treatment effects (with 95% confidence intervals) and will be adjusted for important covariates (prior fracture history and age). The final statistical analysis will include data from patients from the control arm and intervention arm(s) continuing beyond the interim analysis from both the first and second stages of the trial. The analysis needs to allow for the adaptation made at the interim analysis in order to ensure that the probability of a type I error (false positive result) is controlled. It is proposed to use the analysis method described by Bretz and colleagues [44], applying a Dunnett correction to the comparisons between the intervention arms and the control arm at the first stage and at the second stage unless an intervention arm is dropped.

Complier-average causal effect will be estimated to assess the impact of adherence on the effect of the interventions.

### Health economics

Mean (and standard deviation) per patient costs and QALYs for the control and for the intervention(s) selected for the full 12-month evaluation through the adaptive trial design will be computed. Modelling will be used to extrapolate clinical events, costs and QALYs beyond the 12-month trial follow-up. Incremental analyses will be performed, with differences in costs and QALYs between alternatives calculated, and an incremental cost(s) per QALY gained will be estimated. Uncertainty around the cost per QALY value(s) will be quantified using non-parametric bootstrapping. Results from the economic evaluation will identify the intervention with the greatest probability of being cost-effective given the NHS’s willingness to pay for additional health gain.

### Timeline

The trial is funded to run over a period of 51 months and commenced in January 2013. The final follow-up visit for the final participant is projected to be completed by the end of month 42 (that is, by June 2016). Data analysis, economic analysis and report writing will be from month 45 onwards.

## Discussion

Osteoporosis and vertebral fracture have a considerable impact on an individual’s health-related QoL due to pain, limitations in activity and social participation and altered mood. The need to develop effective treatment approaches for osteoporosis and to decrease the health burden of associated vertebral fractures is an important clinical and research objective. This pragmatic RCT will provide high quality evidence of the longer term clinical and cost effectiveness of two physiotherapy-based interventions. Overall, there is evidence of proof of concept that both manual therapy and exercise interventions can be beneficial for this patient group. However, currently there are questions about compliance with exercise programmes and whether a positive effect can be seen at levels of intervention intensity that are deliverable within current NHS resources. This trial design allocates equivalent physiotherapist contact time to participants in each intervention group and broadly reflects current outpatient physiotherapy resources within the NHS which should assist with delivery and generalisability of any findings.

Our study offers a novel approach in using an adaptive trial design to assess the efficacy of a rehabilitation intervention. At the outset the plan is to conduct a multi-centred, three-arm RCT with blinded assessments. The justification for using an adaptive design is that this is a new and emerging area of clinical practice. Instead of offering a “black box” of combined manual and exercise therapies, we have a chance to estimate the effects of these treatment strategies in isolation. Each is indicated for adults with symptomatic osteoporotic vertebral fracture. There are some reported concerns with the efficacy and safety of spinal mobilisation [10,11], thus there is merit in testing manual therapy in isolation against an exercise-based intervention. Additionally, spinal mobilisations are the most expensive physiotherapy modalities to deliver as they cannot be self-administered, and require individual sessions. Using an adaptive design allows the trial to be adapted to a two-arm trial, with resulting increased power, should one of the intervention arms be assessed as ineffective at interim analysis. If neither intervention shows benefit, the trial would be stopped early using futility rules. We believe that this adaptive design has merits over testing a multi-modal programme of combined exercise and manual treatments where it is difficult to evaluate the efficacy of individual components. There would also be significant concerns about the safety and cost of some aspects of the multi-modal programme, that make isolating the interventions an attractive option in terms of new knowledge gained.

Importantly, the pragmatic approach offered in this trial allows programmes in both intervention arms to be individualised with regard to the content and intensity level of the exercise or manual therapy technique. This makes it less artificial than many RCT intervention designs and thus much closer to normal clinical practice.

Our study is the first large-scale RCT to investigate the effect of different physiotherapy management strategies – exercise or manual therapy intervention for patients with osteoporosis and vertebral fracture. We plan to recruit 600 patients to the trial. Even if the adaptive design and interim analysis rules proved that neither intervention were effective and the trial was stopped on the grounds of futility, it will still be the largest RCT conducted of physiotherapy and osteoporosis. Strengths of the study design are the pragmatic nature of treatment delivery by practicing physiotherapists in NHS physiotherapy clinics across the UK, as well as the reproducibility of both the physiotherapy intervention programmes. These features will improve the ability to translate the findings into a standard practice and enable future delivery of the intervention to be given within existing NHS resource levels and the constraints of a publically funded health system.

In addition to this paper, updated versions of the protocol if amended throughout the trial will be available on the trial website (http://www.ndorms.ox.ac.uk/clinicaltrials/prove) and will follow SPIRIT 2013 guidelines [45].

## Trial status

The first patient was randomised to the trial in September 2013. Recruitment for the study is ongoing.

## Abbreviations

BMD: bone mineral density; IDMC: independent data monitoring committee; NHS: National Health Service; PROVE: Physiotherapy Rehabilitation for Osteoporotic Vertebral Fracture; QALY: quality-adjusted life year; QoL: quality of life; RCT: randomised controlled trial; TLS: Timed Loaded Standing.

## Competing interests

The authors declare that they have no competing interests.

## Authors’ contributions

KLB, SL and MN conceived the project. KLB procured the project funding and is the Principal Investigator for the trial. KLB, SL, MKJ and MN assisted with protocol design. KLB and MN designed the physiotherapy programme. CML developed the qualitative part of the trial design and will lead on the delivery of this aspect of the trial. NS performed the sample size calculation, developed the adaptive trial simulations to inform the interim analysis decision rules and will supervise all statistical aspects of the trial. HC designed and will lead on the health economic evaluation of the trial. VG, KLB and MN wrote the procedures manual, and the information and trial education materials. KLB wrote the first draft of this manuscript. All authors participated in the trial design, provided feedback on drafts of this paper and read and approved the final manuscript.
